# Glutamate and asparagine cataplerosis underlie glutamine addiction in melanoma

**DOI:** 10.18632/oncotarget.3132

**Published:** 2015-02-28

**Authors:** Boris Ratnikov, Pedro Aza-Blanc, Ze'ev A. Ronai, Jeffrey W. Smith, Andrei L. Osterman, David A. Scott

**Affiliations:** ^1^ Sanford-Burnham Medical Research Institute, La Jolla, California 92037, USA

**Keywords:** Melanoma, Glutaminolysis, Cataplerosis, Stable isotopes

## Abstract

Glutamine dependence is a prominent feature of cancer metabolism, and here we show that melanoma cells, irrespective of their oncogenic background, depend on glutamine for growth. A quantitative audit of how carbon from glutamine is used showed that TCA-cycle-derived glutamate is, in most melanoma cells, the major glutamine-derived cataplerotic output and product of glutaminolysis. In the absence of glutamine, TCA cycle metabolites were liable to depletion through aminotransferase-mediated α-ketoglutarate-to-glutamate conversion and glutamate secretion. Aspartate was an essential cataplerotic output, as melanoma cells demonstrated a limited capacity to salvage external aspartate. Also, the absence of asparagine increased the glutamine requirement, pointing to vulnerability in the aspartate-asparagine biosynthetic pathway within melanoma metabolism. In contrast to melanoma cells, melanocytes could grow in the absence of glutamine. Melanocytes use more glutamine for protein synthesis rather than secreting it as glutamate and are less prone to loss of glutamate and TCA cycle metabolites when starved of glutamine.

Melanoma is the deadliest form of skin cancer and is difficult to treat once it metastasizes. Immunotherapy is significantly improving long-term survival in a subset of melanoma patients [1], and new drugs, particularly those that target mutant BRAF found in about 50% of melanoma, have provided some hope, but resistance and relapse are typically encountered [2]. Therefore there is still an urgent need for the discovery and development of novel targets for melanoma therapy. Among the prominent areas where such targets are sought is metabolism, as it is well established that cancers have divergent metabolism compared to normal tissues. In previous studies on melanoma cells, we found a typical highly glycolytic (glucose-to-lactate) metabolism [3]. Additionally, though, glutamine usage by melanoma cells was increased compared with melanocytes, the normal cellular precursors of melanoma [3]. This appeared to be a general phenomenon regardless of the melanoma mutational background. Although glucose uptake was 5–10-fold greater than glutamine uptake, most of the glucose was converted to lactate, and glutamine was therefore demonstrated to provide essential input into the tricarboxylic acid (TCA) cycle [3].

A glutamine requirement for the proliferation of many cell types is well established [4, 5] and certain tumor or oncogene-modified cell lines undergo apoptosis when deprived of glutamine [6–8]. The metabolism of glutamine begins by its conversion to glutamate by glutaminase, or other amidases which have various functions in biosynthetic pathways [9]. Deamination of glutamate yields α-ketoglutarate, an intermediate in the TCA cycle, and thereby glutamine acts as an anaplerotic substrate [10], contributing to the maintenance of pools of carboxylic acids in the TCA cycle and sustaining cellular oxidative phosphorylation (Figure 1A). This use of glutamine as an energy substrate is known as glutaminolysis, by analogy to glycolysis [11]. Additionally, the carbon contributed to the TCA cycle by glutamine can be used in biosynthetic reactions [12] (Figure 1A), and the processes of glutaminolysis and biosynthesis can run in parallel. Anaplerosis and cataplerosis (green and red arrows respectively in Figure 1A) must be balanced to maintain the TCA cycle in equilibrium [13]. Studies on tumor cells using ^13^C-glutamine have confirmed the use of glutamine as an anaplerotic substrate [3, 14–18], and have outlined some of the cataplerotic roles of carbon derived from glutamine, including use for fatty acid synthesis under hypoxia [3, 19–21] and export from the mitochondrion as aspartate and generation of NADPH (which maintains cellular redox state) via malic enzyme activity [16]. It is also well known that tumor or normal cells fed with glutamine will secrete glutamate [22, 23], and the secretion of some partially-^13^C glutamate from cells labeled with universally-^13^C-glutamine, which indicates TCA cycle-origin (Figure 1B), has been reported [15]. Glutamate derived from the TCA cycle contributes to cataplerosis and therefore provides a potential route for loss of TCA cycle metabolites, a process which has been observed in glutamine-starved cells undergoing apoptosis [6].

**Figure 1:**
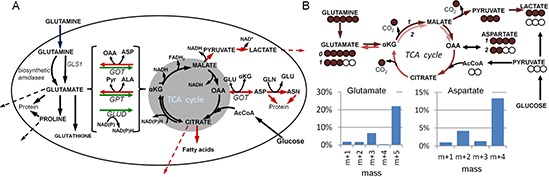
Maps of glutamine metabolism **(A)** Anaplerotic conversion of glutamate to α-ketoglutarate (αKG) is highlighted by green arrows (alternate enzymatic reactions for this step are contained within the brackets); cataplerotic reactions are shown by red arrows; TCA cycle is within the shaded area. Dashed lines indicate secretion of metabolites. Genes for key steps are shown: gls1, glutaminase-1; got, glutamate-oxaloacetate transaminase (aspartate aminotransferase); gpt, glutamate-pyruvate transaminase (alanine aminotransferase); glud, glutamate dehydrogenase. Other abbreviations: Pyr, pyruvate; OAA, oxaloacetate; AcCoA, acetyl-coenzyme-A. **(B)** Using ^13^C-labeling to quantify glutaminolysis. Metabolic trafficking from universally ^13^C-glutamine is indicated by brown arrows, with ^13^C atoms, including ^13^C lost as CO_2_, indicated by brown circles, and recombination with unlabeled (open circles) acetyl-CoA in the TCA cycle. Routes to glutamate, aspartate and lactate are shown, as well as example mass profiles of cellular glutamate and aspartate. The x axis designations of “m+1”, “m+2”, etc., indicate metabolite mass greater than the unlabeled (no ^13^C) mass of “m+0”, and together indicate the distribution of ^13^C label. ^13^C-Glutamine is deamidated to (m+5) glutamate. This is not glutaminolysis, as no energy is produced. But glutamate can then be converted to α-ketoglutarate and circuit the TCA cycle. After one circuit, two carbons are lost as CO_2_ and replaced by unlabeled acetyl-CoA. This results in m+3 glutamate. Further circuits of the TCA cycle result in exchange of more ^13^C carbon. Similarly for aspartate, the initial pass through the TCA cycle yields m+4 aspartate (one carbon lost as CO_2_), and another circuit through the cycle yields m+2 aspartate (three carbons lost as CO_2_). Glutaminolysis can be quantified in terms of CO_2_ production by comparing the ^13^C content and quantities of metabolites after ^13^C-glutamine labeling with the amount of ^13^C-glutamine taken up by cells.

Despite these numerous studies, there are significant gaps in our knowledge of glutamine metabolism in tumor cells. In particular, apart from one seminal example in which a glioma cell line was found to convert 60% of the consumed glutamine into lactate [24], there have been few studies to define exactly how the carbon content of glutamine is distributed between biosynthesis, glutaminolysis, or secretion as glutamate or other metabolites. Here, quantitative audit of glutamine utilization by melanoma cells demonstrated that TCA cycle-derived glutamate, not lactate, was generally the major product of glutaminolysis, irrespective of the cells' oncogenic background. Additionally, experiments where glutamine was limited, but alternate metabolites were added, supported a role for glutamine in maintaining the TCA cycle but also identified aspartate and asparagine synthesis as important cataplerotic pathways which contribute to “glutamine addiction” in melanoma. In contrast to melanoma cells, melanocytes could grow in the absence of glutamine, and exhibited metabolic differences consistent with less glutaminolysis.

## RESULTS

### Melanoma cells require glutamine for asparagine synthesis and to maintain the TCA cycle

**Figure 2:**
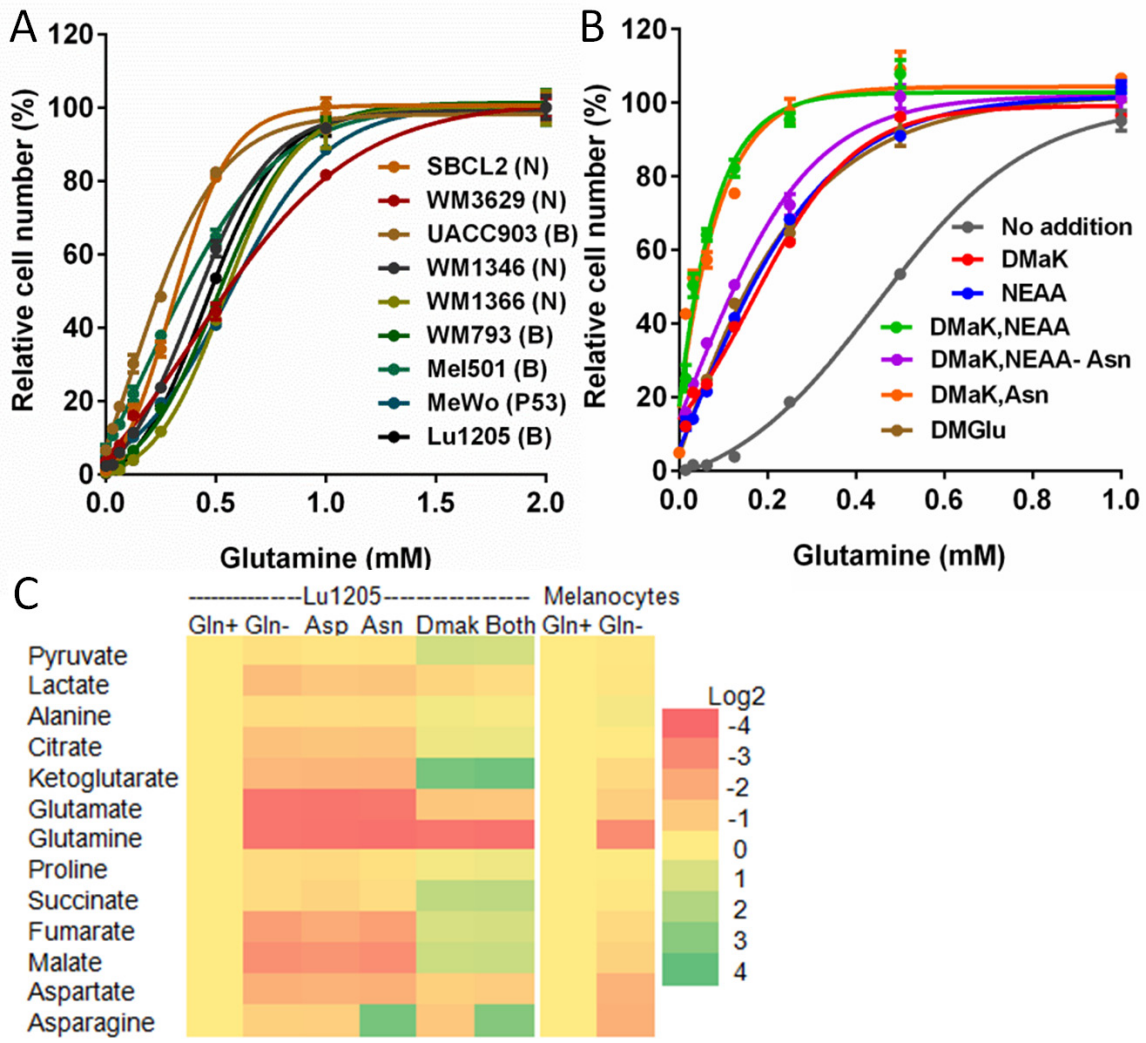
Glutamine is required for growth and used to sustain TCA cycle metabolite levels and for aspartate and asparagine synthesis **(A)** Effects of titrating glutamine in DMEM on growth of melanoma cells lines. Mutated oncogenes in cell lines are designated: (B) BRAF; (N) NRAS; (P53) TP53. **(B)** Growth of Lu1205 cells in DMEM with varied glutamine and supplementation with 3 mM DMaK, 0.1 mM NEAA, 0.1 mM asparagine (Asn), or 5 mM DMGlu. “NEAA-Asn” indicates addition of a reconstituted NEAA mixture lacking asparagine. Growth is shown relative to growth in medium containing 2 mM glutamine (Mean ± SEM of *N* = 3). (**C**) Changes in metabolite pools in Lu1205 cells or melanocytes after 6 h in glutamine-free medium(Gln-), relative to cells with 2 mM glutamine (Gln+). Lu1205 Gln− cultures were supplemented as shown with 3 mM DMaK, 0.1 mM Asn, 0.1 mM aspartate (Asp), or (“Both”) DMaK and Asn. Source data for part (C) are shown in Supplementary Dataset 1.

We tested the glutamine dependence of nine melanoma lines with different oncogenic drivers (4 mutant BRAF, 4 mutant NRAS, 1 mutant p53). All required at least 1 mM glutamine for maximal growth (Figure 2A) and there was no proliferation in the absence of glutamine (Figure S1A, S1B), while most cell lines could grow in the absence of glucose (Figure S1C). In contrast, growth of melanocytes was similar with or without glutamine (Figure S1D). As melanocytes were grown in a melanocyte-specific medium, we checked growth of Lu1205 melanoma cells in this medium and confirmed that growth was substantially inhibited by the absence of glutamine (Figure S1E).

To test whether glutamine could be replaced by other nutrients, we measured the effects of cell-permeable di-methyl derivatives of α-ketoglutarate (DMaK) or glutamate (DMGlu), or non-essential amino acids (NEAA culture supplement) on the growth of Lu1205 cells. Each of these reduced the amount of glutamine required for maximum growth by 50%, to 0.5 mM (Figure 2B). Other potential biosynthetic products of glutamine utilization, uridine, hypoxanthine, glutathione and hemin, did not reduce the glutamine requirement (Figure S2A). Addition of NEAA and DMaK together further reduced the concentration of glutamine required for optimal growth, to 0.25 mM (Figure 2B). Analysis of the NEAA requirement showed that addition of asparagine alone could substitute for NEAA, and that adding the other components of NEAA without asparagine only slightly reduced the glutamine requirement (Figure 2B). This result indicates that, in the absence of external asparagine, asparagine synthesis accounted for up to 50% of the glutamine used in these cells. Similar improvement in growth in low-glutamine medium was observed by addition of asparagine or DMaK to cultures of six other melanoma cell lines (Figure S2B). We confirmed that glutamine could contribute to the carbon backbone of asparagine by labeling Lu1205 cells with ^13^C-glutamine and determining synthesis of asparagine and its immediate biosynthetic precursor, aspartate, from glutamine (Figure S2C).

As another approach to investigating the roles of glutamine in melanoma cells, we deprived Lu1205 cells of glutamine (with or without DMaK, asparagine or aspartate) and measured changes in cellular metabolite levels after 6 h (Figure 2C, Supplementary Dataset 1). Glutamine deprivation resulted in reductions in glutamine, TCA cycle metabolites, and metabolites peripheral to the TCA cycle - glutamate, aspartate, asparagine, pyruvate and lactate. Addition of DMaK restored, or enhanced, levels of most metabolites depleted with glutamine removal, with the exception of glutamine and asparagine. Addition of 0.1 mM aspartate, a concentration five-fold greater than physiological concentrations of aspartate in the blood [25], did not reverse the effects of glutamine deprivation. Asparagine (0.1 mM) was also without effect on the depletion of most metabolites, but elevated the level of cellular asparagine by > 10-fold. The effect of combined DMaK and asparagine supplementation was additive. These results indicated, first, that glutamine deprivation leads to loss of metabolites in the TCA cycle and associated pathways, and are consistent with its role as an anaplerotic substrate. Secondly, aspartate uptake was weak and insufficient to compensate for loss of glutamine. Thirdly, exogenous asparagine restored cellular asparagine pools, but due to an apparent lack of asparaginase activity, asparagine was not converted to aspartate and TCA cycle metabolites. These conclusions were supported by labeling cells using 0.1 mM ^13^C-aspartate or ^3^C-asparagine. ^13^C-aspartate addition to cultures only weakly labeled aspartate or TCA cycle metabolites (Figure S2D). In cells provided with ^13^C-asparagine, cellular asparagine was labeled, but lack of labeling of aspartate or TCA cycle metabolites confirmed that there was negligible conversion of asparagine into these metabolites (Figure S2E). In contrast to these melanoma cells, glutamine deprivation of melanocytes for 6 h resulted in much less loss of cellular glutamate (45% loss in melanocytes versus 92% loss in Lu1205) and TCA cycle metabolites (maximally, 41% loss of malate in melanocytes versus 85% loss in Lu1205, Figure 2C, Supplementary Dataset 1). This resilience to glutamine depletion likely contributes to the ability of melanocytes to grow in the absence of glutamine.

### TCA cycle-derived glutamate is the major end product of glutaminolysis in melanoma cells

**Figure 3:**
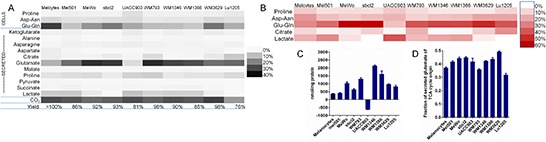
Distribution of carbon from glutamine in melanoma cells and melanocytes emphasizes role of glutamate secretion **(A)** Yield of carbon from glutamine in total cellular amino acids (including protein), in secreted metabolites, and estimated fraction lost as CO_2_ after culture of cells for 72 h in medium containing U-^13^C-glutamine. Abbreviations: Melcytes: melanocytes; Asp-asn: combined cellular aspartate and asparagine; Glu-gln: combined cellular glutamate and glutamine. **(B)** Metabolites associated with calculated CO_2_ production (products of glutaminolysis). CO_2_ production was estimated as the sum of “missing” ^13^C in partially labeled end-point metabolites plus CO_2_ necessarily lost between glutamine and the measured metabolites. Metabolites associated with at least 5% of estimated CO_2_ production are shown, with Asp-asn representing combined cellular and secreted aspartate and asparagine, and Glu-gln, combined cellular glutamate and glutamine and secreted glutamate. (**C**) UACC903 cells show net uptake of glutamate, unlike other melanoma cell lines, which secrete glutamate. (**D**) Fraction of secreted glutamate of TCA-cycle origin (partially ^13^C-labeled). For UACC903 cells, there was no net secretion, but there was exchange of labeled glutamate into the medium (Mean ± SEM of *N* = 3). Source data for parts (A, B) are shown in Supplementary Dataset 2.

To establish more generally how glutamine is used, we quantified the fate of carbon derived from glutamine in melanoma cells or melanocytes. Cells were cultured for 72 h with universally-^13^C-labeled glutamine and the major metabolites into which ^13^C accumulated – total cellular (protein-digested) amino acids and secreted metabolites in culture medium - were quantified and their degree of ^13^C-labeling was determined using GC-MS. Quantification of ^13^C in metabolites, relative to the amount of ^13^C-glutamine taken up by cells, allowed us to calculate the distribution of carbon from glutamine into cellular and extracellular metabolites (Figure 3A; see also Supplementary Dataset 2). We also estimated the amount of carbon from glutamine converted to CO_2_, based on quantities and mass isotopomer distributions of ^13^C-labeled metabolites (Figure 3A, 3B; see Methods and Figure 1B for details). The combination of cellular amino acids, secreted metabolites and CO_2_ on average accounted for 90% of glutamine usage by melanoma cells or melanocytes (Figure 3A). Secreted glutamate generally represented the largest destination for carbon from glutamine – as much as 35% of the total in WM793 cells. Exceptions were melanocytes, where 32% of the carbon from glutamine was assimilated into protein as glutamate or glutamine but only 12% was secreted as glutamate, and UACC903 cells, where extracellular glutamate was a minor product of glutamine (5% of total glutamine carbon). Consistent with this, UACC903 cells, unlike other cell lines, took up glutamate rather than secreting it (Figure 3C).

The fraction of inferred total CO_2_ production associated with different end-products of glutamine (Figure 3B) indicates the relative importance of these metabolites as products of glutaminolysis. For most cell lines, the major product of glutaminolysis was glutamate. Importantly, this indicated that this glutamate was not simply the deamidation product of glutamine but had traversed the TCA cycle, replacing carbon released as CO_2_ with unlabeled carbon from acetyl-CoA before reaching its final destination (Figure 1B). Of the glutamate that was secreted, 30–50% was partially ^13^C-labeled, indicating that it originated from the TCA cycle (where ^13^C atoms were exchanged; Figure 3D). This result is significant because it shows that glutamate secretion is a conduit through which TCA cycle metabolites could be lost during glutamine deprivation (as in Figure 2C). In the case of the UACC903 cell line, where there was no net glutamate secretion, lactate was a more significant product of glutaminolysis than glutamate. In general, though, lactate labeling from glutamine and the associated CO_2_ production were low, and were undetectable in two cell lines, Lu1205 and sbcl2 (Figure 3A, 3B).

### Roles of aminotransferases and glutamate dehydrogenase in anaplerosis and cataplerosis

The above results pointed to the importance of two input/output reactions on either side of the TCA cycle for glutamine metabolism. The conversion of glutamate to α-ketoglutarate is an anaplerotic reaction, and, in reverse, a cataplerotic reaction for the production of TCA-cycle-derived glutamate (Figure 1). Potential enzymes for this reaction are mitochondrial alanine aminotransferase (GPT2), aspartate aminotransferases (cytosolic GOT1 and mitochondrial GOT2), and glutamate dehydrogenase (GLUD, Figure 1A). On the other side of the TCA cycle, the GOT2-catalyzed cataplerotic oxaloacetate-to-aspartate reaction is required for aspartate and thence asparagine synthesis. To test the role of aminotransferases in glutamine utilization, we used the general mechanism-based inhibitor aminooxyacetate (AOA). AOA in the absence of amino acid supplements potently inhibited growth (Figure S3A), but a combination of 0.1 mM alanine or NEAA, plus ≥ 2.5 mM aspartate or dimethyl-asparate substantially reversed AOA inhibition of growth of Lu1205 cells (Figure S3A, S3B) or other melanoma cell lines (Figure S3C). The requirement for supra-physiological levels of aspartate further indicated poor aspartate uptake in these cells.

**Figure 4:**
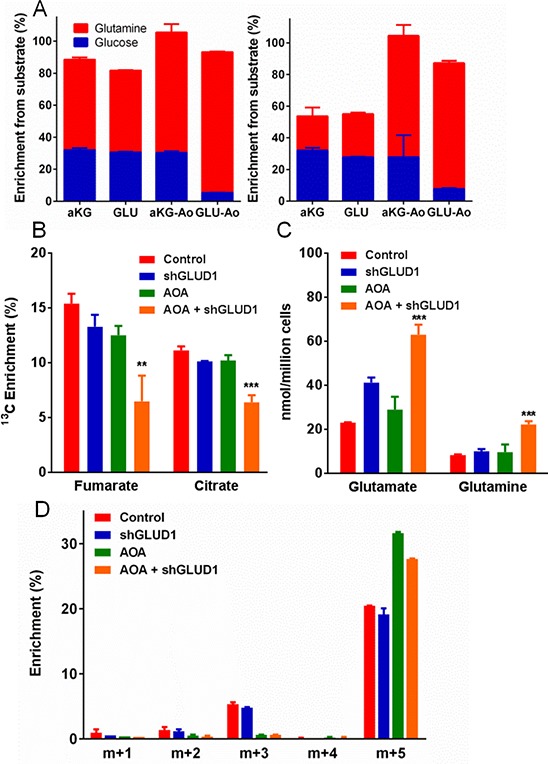
Testing the metabolic functions of aminotransferases and glutamate dehydrogenase (GLUD1) in Lu1205 cells **(A)** Comparative enrichment of α-ketoglutarate or glutamate from glucose or glutamine in Lu1205 cells (left panel) or melanocytes (right panel) ± 0.5 mM AOA. (**B–D**) Effects of combined AOA treatment and *GLUD1* knockdown on metabolism and growth. **(B)** Labeling of TCA cycle metabolites fumarate and citrate from ^13^C-glutamine. (**C**) Quantities of cellular free glutamate and glutamine. (**D**) Mass distribution of secreted glutamate (corrected for natural labeling and ^13^C-glutamine impurity). (Mean ± SEM of *N* = 3 for all, except mean ± range of *N* = 2 for D). **p < 0.05, ***p < 0.01 by Student's unpaired *t*-test.

To assay the roles of aminotransferases in controlling flux into and out of the TCA cycle, we labeled Lu1205 cells or melanocytes with either ^13^C-glucose or ^13^C-glutamine, and compared the effects of AOA treatment on the ^13^C-enrichment of glutamate or α-ketoglutarate. In untreated Lu1205 cells (Figure 4A, left panel), cellular α-ketoglutarate and glutamate were both approximately 30% derived from glucose and 50% derived from glutamine, demonstrating equilibration in the interconversion of these metabolites. In AOA-treated cells, ^13^C input from glutamine (via glutamate) to α-ketoglutarate was maintained or increased, indicating active glutamate to α-ketoglutarate conversion. However, ^13^C input from glucose (via α-ketoglutarate) to glutamate was much reduced, indicating that the α-ketoglutarate to glutamate reaction was inhibited (see Figure 1A). Therefore, these cells convert α-ketoglutarate to glutamate largely using AOA-sensitive aminotransferases, but they retain the capacity to convert glutamate to α-ketoglutarate even when aminotransferases are inhibited (apparently due to the contribution of oxidative deamination by GLUD1 as discussed below).

In melanocytes (Figure 4A, right panel), the same type of analysis revealed a substantially lower relative contribution of glutamine to the synthesis of α-ketoglutarate and glutamate (in the absence of AOA). Notably, there was not a corresponding increase in the fraction of these metabolites derived from glucose, which implied that other unlabeled carbon sources were contributing to their synthesis. After addition of AOA, though, the derivation of these metabolites from glucose or glutamine closely matched the Lu1205 results, and the contribution from unlabeled sources was largely eliminated. This suggests that melanocytes use AOA-sensitive aminotransferases to convert other amino acids (e.g., branched chain amino acids) into TCA cycle metabolites, thus making melanocytes less dependent on anaplerotic utilization of glutamine.

The most likely candidate for the AOA-resistant unidirectional deaminating activity was glutamate dehydrogenase, and to test this, we used Lu1205 containing two different inducible glutamate dehydrogenase (GLUD1) knockdown constructs, which reduced the expression of *GLUD1* by ~90% as indicated by both qPCR and measurement of glutamate dehydrogenase activity (Figures 4B–4D and S3D–G). Labeling of TCA cycle metabolites by ^13^C-glutamine was not reduced in GLUD1 knockdown cells, except with the addition of AOA, indicating that both aminotransferase and glutamate dehydrogenase reactions contribute to anaplerotic flux from glutamine into the TCA cycle (Figure 4B). GLUD1 knockdown increased cellular glutamate pools, consistent with less conversion of glutamate to α-ketoglutarate, and this effect was further enhanced by the addition of AOA (Figure 4C). Only combined GLUD1 knockdown and AOA treatment increased cellular glutamine (Figure 4C). Meanwhile, analysis of ^13^C-labeling of glutamate in medium from these cultures indicated that GLUD1 knockdown had no effect on the labeling pattern of glutamate (Figure 4D), but AOA treatment eliminated partially-labeled (m+1, m+2, m+3) glutamate and increased the proportion of fully labeled (m+5) glutamate. This indicated that, with AOA treatment, glutamate could be synthesized directly from glutamine (m+5 labeling), but its synthesis from the TCA cycle (via partially-labeled α-ketoglutarate) was blocked. These results confirmed that GLUD1 and aminotransferases were together responsible for glutamate-to-α-ketoglutarate conversion in these cells, but the reverse reaction, required for glutamate-generating glutaminolysis (Figure 1B), was mediated only by aminotransferases.

To test the roles of specific aminotransferases, we knocked down *GOT1, GOT2* and *GPT2*. Knockdown of *GPT2* totally inhibited alanine secretion (Figure S4A), and *GOT2* knockdown led to an accumulation of glutamate (Figure S4B), implying that the net flux from both these reactions was in the direction of α-ketoglutarate production (Figure 1A). Knockdown of *GOT1* but not *GOT2* led to an accumulation of aspartate (Figure S4C), confirming that GOT1 was mainly responsible for α-ketoglutarate-to-glutamate and aspartate to oxaloacetate conversion (the classic role of GOT1 in the malate-aspartate shuttle [12]). The knockdown of any individual aminotransferase (*GOT1, GOT2* or *GPT2*), unlike AOA treatment (Figure 4D), did not affect the labeling pattern of secreted glutamate originating from ^13^C-glutamine (shown as the m+3/m+5 ratio, Figure S4D). These results implied that no one aminotransferase fully controlled the glutamate-α-ketoglutarate reaction in either direction.

## DISCUSSION

This study leads to the following salient conclusions: (i) unlike melanocytes, melanoma cells cannot grow without millimolar quantities of glutamine, and correspondingly melanoma cells consume up to 7-fold more glutamine than melanocytes [3]; (ii) Glutamate/α-ketoglutarate or asparagine are the only metabolites that substantially reduce the glutamine requirement of melanoma cells (by ~50% or ~75% if used in combination); (iii) Glutamine, via glutamate, is a major anaplerotic substrate, entering the TCA cycle by combined action of aminotransferases (GOT2 and GPT2) and glutamate dehydrogenase (GLUD1); (iv) Paradoxically, glutamate is also the primary cataplerotic output from the TCA cycle (Figure 1A), and the major endpoint of (CO_2_-producing) glutaminolysis; (v) Asparagine is not metabolized and is apparently only used for protein synthesis. Its precursor aspartate is an essential cataplerotic product as it cannot be salvaged at physiological concentrations.

One objective of this study was to determine how the metabolism of glutamine differs in melanoma cells versus melanocytes. Glutamine was essential for the growth of melanoma cells of diverse oncogenic backgrounds (mutated BRAF, NRAS or p53). Comparative analysis of these cell lines suggests that glutamine addiction is probably independent of the initial oncogenic insult, and could be regulated by factors that are commonly downstream of many oncogenes. Indeed, where there were differences in melanoma glutamine metabolism, as in the case of glutamate secretion by UACC903 cells, it was individual cell lines that were different and not a whole oncogenic class of cell lines. The resistance of melanocytes to glutamine deprivation compared to melanoma cells may be based on a combination of glutamine conservation and use of other carbon sources. First, they use a larger fraction of glutamine for protein synthesis and secrete less as glutamate (Figure 3A). Second, when deprived of glutamine, the loss of metabolites from the TCA cycle is much less in melanocytes than Lu1205 cells (Figure 3C). Third, ^13^C-labeling combined with AOA treatment indicated a large fraction of glutamate/α-ketoglutarate in melanocytes (but not Lu1205 cells) is probably derived from amino acid(s) other than glutamine (Figure 4A).

Previous work on glioma cells [24] established that, in at least some cases, glutaminolysis leads predominantly to production of lactate. However, we determined that lactate was a relatively minor product of glutaminolysis in melanoma cells: 0–9% of glutamine carbon taken up by cells was destined for secreted lactate (Figure 3A; Supplementary Dataset 2) and only in UACC903 cells was lactate the main glutaminolysis product (Figure 3B). Whether this flux to lactate represents a significant pathway for NADPH synthesis and therefore maintenance of redox balance in any of these cells, as recently shown in pancreatic cancer cells [16], is an open question, as the importance of this pathway for NADPH production depends on the relative activity of other routes for NADPH synthesis (e.g., the pentose phosphate pathway).

In general, glutamine-consuming tumor cells secrete glutamate [26]. Recent papers using ^13^C tracers and flux analysis [14, 27] calculated rates of glutamate secretion and glutamine uptake but did not specifically calculate how much of the carbon in the secreted glutamate is derived from glutamine (as opposed to other carbon sources). Here, we used ^13^C tracing to determine whether the secreted glutamate comes straight from glutamine (remaining fully ^13^C-labeled), or has circuited the TCA cycle (partial ^13^C-labeling indicating loss of ^13^CO_2_ and acquisition of unlabeled carbon). This is important as it measures the degree to which glutamate is an end-product of glutaminolysis in the TCA cycle. Additionally, by measuring the proportion of secreted glutamate that is only partially ^13^C-labeled (versus the fully-^13^C-labeled input glutamine), we can measure how much of the secreted glutamate is of TCA cycle origin. This is of relevance for growth inhibition upon glutamine deprivation (as we observe for melanoma cell lines, Figures 2A and S1), as removing the glutamine input to the TCA cycle leads to loss from cells of TCA cycle metabolites (Figure 2C).

It may seem obvious, given the well-known reversibility of aminotransferase-mediated reactions, that secreted glutamate could be derived from the TCA cycle. What is less obvious is the relative contribution of aminotransferases versus glutamate dehydrogenase to the inter-conversion of α-ketoglutarate and glutamate in cells. As our results in Figure 4A indicate, glutamate dehydrogenase in these cells only mediates the unidirectional conversion of glutamate to α-ketoglutarate, whereas PLP-dependent aminotransferases (such as GOT and GPT) can drive this transformation in both directions (Figure 1A). The balance between glutamate dehydrogenase and aminotransferase activity in tumor cells has been shown to vary as demonstrated by changing KRAS expression in pancreatic cancer cells [16], so the actual direction of flux between glutamate and α-ketoglutarate may vary depending on cell type.

Various data indicate melanoma cells rely on aspartate synthesis rather than uptake. The normal physiological (blood) concentration of aspartate is 20 μM [25], but addition of 100 μM aspartate did not reverse aspartate depletion following glutamine deprivation (Figure 2C), and the same concentration of ^13^C-labeled aspartate only weakly labeled cellular aspartate (Figure S2D). In most melanoma lines studied, AOA was a potent inhibitor of growth even in the presence of NEAA, which includes 100 μM aspartate. Inhibition was only reversed by adding ≥ 2.5 mM aspartate or dimethyl-aspartate (Figure S3). Aspartate synthesis is therefore of interest as a point of vulnerability in melanoma. In studies on other cancers, the role of GOT, but not aspartate synthesis per se, has been considered. In breast cancer [28], siRNA was used against GOT1, which, as we confirm in Figure S4, is functional physiologically in the direction of aspartate to oxaloacetate conversion rather than aspartate synthesis. Likewise, in pancreatic cancer [16], the focus was on GOT1 in a pathway leading to NADPH production by malic enzyme.

As aspartate is a biosynthetic precursor to asparagine, inhibition of aspartate synthesis could be usefully potentiated by combination with asparaginase treatment, which is a long-established therapy for leukemia [29]. Asparaginase treatment would drive melanoma cells to increase asparagine synthesis from aspartate and glutamine (Figure 1A). Importantly, the microbial asparaginases used for leukemia have activity on glutamine as well as asparagine [30], and therefore could reduce exogenous glutamine supply at the same time as demand for glutamine increased. Glutamine deprivation has been demonstrated to induce asparagine synthetase expression via a GCN2/ATF4 signaling pathway [31], which ironically would further increase demand for glutamine. Indeed, it has recently been shown that SF188 glioma cells deprived of glutamine turn on asparagine synthetase expression even with the addition of external asparagine [32].

In essence, “glutamine addiction” is an enhanced requirement for glutamine, the restriction of which threatens cell survival. In this paper, we have tracked the major uses of glutamine in melanoma cells. The high-demand of glutamine carbon for certain reactions may limit its availability for other minor, yet vital, roles, including use by biosynthetic amidases (for purines, NAD, hexosamines, etc.) and, as glutamate, for glutathione. All of these functions may contribute to the glutamine requirement of melanoma *in vivo*.

## METHODS

### Cell lines and media

The melanoma cell lines used were described previously [33]. The identified tumorigenic mutations in these lines are: Lu1205, WM793, mel501, UACC903, and WM3629: BRAF mutation; WM1346, WM1366, and sbcl2: NRAS mutation; MeWo: p53 mutation. Primary human dermal melanocytes were a kind gift of Dr. Mikhail Nikiforov, Roswell Park Cancer Institute. Melanoma cells were routinely grown in DMEM (high glucose) supplemented with 4 mM Glutamine (Cellgro), 100 U/mL penicillin, 100 μg/mL streptomycin and 10% (v/v) fetal bovine serum (Hyclone) (FBS) in a humidified incubator, in the presence of 5% CO_2_, at 37°C. Melanocytes were grown in Dermal Cell Basal Medium supplemented with the Melanocyte Growth Kit (ATCC). For glucose or glutamine deprivation and AOA-inhibition experiments, melanoma cells were cultured in glucose- and glutamine-free DMEM (Sigma), supplemented with 10% dialyzed FBS (Gibco) for 72 h. Control cultures contained 2 g/L glucose and 2 mM L-glutamine, except as noted. AOA was added to cultures at 0.5 mM, except as noted. Glutamine deprivation in melanocytes was assayed by omitting the glutamine component of the melanocyte medium and comparing growth with or without 2 mM glutamine over 240 h (or 72 h for cultures of Lu1205 cells in this medium). Growth was monitored by cell counting following trypsinization, or relative viable cell number was measured after 72 h using the Cell Titer 96 Aqueous Assay (Promega).

### ^13^C-metabolic labeling procedures

For purposes of accounting for glutamine utilization in melanoma, cells were seeded at 100,000/well in 6-well tissue culture plates in DMEM medium. The next day medium was changed to MEM (Cellgro) containing 10% FBS, NEAA, 2 g/L glucose and 2 mM 50% ^13^C-uniformly labeled glutamine (Sigma). Cells were incubated for a further 72 h. To take into account glutamine breakdown or medium evaporation, medium without cells was incubated in parallel. Fractional distribution of ^13^C from glutamine into a metabolite *M* was calculated as [(average per-C ^13^C labeling of *M*) × (number of C in *M*) × (amount of *M* in nmol)]/[(average per-C ^13^C labeling of glutamine) × (number of C in glutamine) × (uptake of glutamine in nmol)]. For labeling in the presence of AOA, cells were incubated for 24 h in this MEM plus NEAA medium or, for glucose labeling, the same medium was used except that the glucose component was 50% ^13^C-uniformly labeled (Sigma) and glutamine was unlabeled. For aspartate or asparagine ^13^C-labeling, the labeling medium was DMEM, 10% dialyzed FBS, 2 g/L glucose, 2 mM glutamine and 100 μM uniformly-^13^C L-asparagine or uniformly-^13^C L-aspartate (both Sigma).

### CO_2_ calculation

To determine how much carbon was converted to CO_2_ during the synthesis of metabolites from (^13^C-) glutamine, first, the CO_2_ that was necessarily generated in the synthesis of (^13^C-) metabolites with fewer carbons than glutamine was counted (e.g., each ^13^C-labeled aspartate with 4 carbons, even if fully labeled, was associated with the production of one molecule of CO_2_ from glutamine with 5 carbons). Secondly, the difference between average fractional per-carbon ^13^C-labeling of a metabolite and the fraction of that metabolite that was ^13^C-labeled to any extent was determined to measure additional ^13^C atoms that were converted to CO_2_ in the production of that metabolite (e.g., in Figure 1B, the M+3 glutamate fraction, with 3 ^13^C-labeled carbons, is associated with the loss of 2 ^13^C-CO_2_ molecules). These calculations assumed that glutamine metabolic pathways were linear and that there was no recombination of ^13^C derived from glutamine. The CO_2_ from glutamine calculated by this method may be underestimated, as glutamine molecules that were fully oxidized leaving no trace of ^13^C in other metabolites are not counted. However, in order to be fully oxidized, the carbons from glutamine would have to circuit the TCA cycle at least three times.

### Gene knockdowns

The effect of gene silencing on metabolic activity of Lu1205 cells was studied using RNAi or stable inducible knockdowns. Expression of *GOT1*, *GOT2* and *GPT2* were knocked down using siRNA. Equimolar (10 nM) mixtures of oligonucleotide duplexes targeted the following sequences: *GOT1* (NM_002079.2): CCUGGGAGUGGGAGCAUAU, GGUAAUGUGAAGACAAUGG, CCACAUCACUGAUC AAAUU, and GGUGCACUUCGAAUUGGA; *GOT2* (NM_001286220.1): GCUACAAGGUUAUCGGUA, GCCAAAAGGGUAGAGUCA, GGGACACCAAUAGCA AAAA, and GGAAUACCUGCCCAUUGGG; *GPT2* (NM_133443.2): CCAUCCAGGUGAAUUACUA, GAUCUUCAUUCCUGCCAAA, and GAAGGCACUUAC CACUUCA. The *GOT1* and *2* siRNA oligos were purchased from Ambion, the *GPT2* siRNA oligo mixture (sc-45647) from Santa Cruz Biotechnology Inc. The non-targeting control siRNA 1 was purchased from Thermo Scientific. RNAi Max reagent (Life Technologies) was used mixed at 1% in OptiMEM with siRNA duplexes for 20 minutes (Life Technologies) followed by 5-fold dilution with growth medium to perform transfections. After 6 hours of incubation transfection media were changed to growth medium of appropriate composition. The efficiency of gene expression knockdowns was verified by western blotting using the following antibodies against: GPT2: SAB1409901 (Sigma) and beta-actin: sc-1618 (Santa Cruz Biotechnology) or by qPCR run on a Stratagene MX3000 analyzer using SYBR green qPCR Supermix (Life Technologies) and the following primers: GOT1 forward: GAAGACAATGGCTGACCGGA; GOT1 reverse: ACTCAACCTGCTTGGGGTTC; GOT2 forward: GGGCTTATATGGTGAGCGTGT; GOT2 reverse: TCGCAAATCTGGGGTGTTCA.

Stable inducible knockdowns were constructed as described [34]. The following oligonucleotides targeting *GLUD1* (NM_005271.3), GTGAATGCCTATAGAAATA (sh#3) or CCATGAAGTGCTAGATAAT (sh#5), and *GOT2* (NM_001286220.1), TCAGGTTCCTCGTGAGAAA, were designed using the algorithm available at http://www.thermoscientificbio.com/design-center and cloned into the lentiviral Tet-pLKO-puro vector (Addgene). The constructs were sequenced and used for transfection of HEK293T cells. Lentiviral particle production and infection of Lu1205 melanoma cells were done per manufacturer's instructions. Following selection at 1 μg/mL puromycin, efficiency of knockdown induced by addition of 100 ng/mL doxycycline (Sigma) for 96 hours was measured by real time qPCR using the primers described above for *GOT2* or, for *GLUD1*, forward: GGAAGCTGCGGCTTAAAAGG; reverse: GTAGCGGTACATGGCCACAA. Additionally, for *GLUD1* knockdown, glutamate dehydrogenase activity was measured in an assay mix containing 0.25 mM NADH, 100 mM sodium phosphate buffer, pH 7.5, 0.1 mM EDTA, 5 mM α-ketoglutarate, 50 mM ammonium chloride, and cell lysate (~1 μg protein in 100 μl volume). Activity was measured in a plate reader at 340 nm and 30°C.

### GC-MS methods

For GC-MS analysis of intracellular metabolites, cells were grown to about 1 million cells/0.5 mg cell protein per culture well. Medium was removed and saved for analysis, cells were washed quickly 3 times with cold PBS, and 0.45 ml cold methanol (50% v/v in water with 20 μM L-norvaline as internal standard) was added to each well. Culture plates were transferred to dry ice for 30 min. After thawing on ice, the methanol extract was transferred to a microcentrifuge tube (the described treatment disrupted cells without the necessity of scraping). Chloroform (0.225 ml) was added, the tube was vortexed and centrifuged at 10,000g for 5 min at 4°C. The upper layer was dried in a centrifugal evaporator and derivatized with 30 μl O-isobutylhydroxylamine hydrochloride (20 mg/ml in pyridine, TCI) for 20 min at 80°C, followed by 30 μl *N*-*tert*-butyldimethylsilyl-*N*-methyltrifluoroacetamide (Sigma or Regis) for 60 min at 80°C. After cooling, the derivatization mixture was transferred to an autosampler vial for analysis.

For digestion of cellular proteins, cells washed as above (while still attached to plates) were lysed in 0.6 ml 10 mM tris-HCl, pH 7.3 containing 1 mM EDTA, 1% Triton X100 and 0.4 mM L-norvaline. A small fraction (20 μl) was dried and digested for 18 h with 200 μl 6N HCl. After drying under nitrogen, the digest was derivatized for GC-MS as above.

For GC-MS analysis of medium, 40 μl of medium was mixed with 0.4 ml cold methanol (50% v/v in water with 20 μM L-norvaline as internal standard). The methanolic extract was counter-extracted with 0.2 ml chloroform, dried, and derivatized as for cell extracts.

GC-MS protocols were similar to those described before [3], except a modified temperature gradient was used for GC: Initial temperature was 130°C, held for 4 min, rising at 6°C/min to 243°C, rising at 60°C/min to 280°C, held for 2 min. Data were corrected for natural ^13^C labeling as before [3]. Metabolites were quantified against varied amounts of standard mixtures run in parallel and data were analyzed using Metaquant [35]. Quantities were corrected for recovery using the L-norvaline internal standard. Glutamine uptake from medium and lactate secretion into medium were measured using a YSI model 7100 enzyme analyzer rather than by GC-MS.

### Reagents

Reagents not listed above were obtained from the following suppliers: dimethyl-aspartate (Bachem); dimethyl-glutamate (Santa Cruz Biotechnology); other reagents were from Sigma.

## SUPPLEMENTARY FIGURES






