# Supplementary material for: Glutamate and asparagine cataplerosis underlie glutamine addiction in melanoma
Source: Oncotarget. 2015 Feb 28;6(10):7379–89. doi: 10.18632/oncotarget.3132 (PMC4480687; doi:10.18632/oncotarget.3132)
Supplement: Supplementary file 1 [file oncotarget-06-7379-s001.pdf]

## SUPPLEMENTARY FIGURES

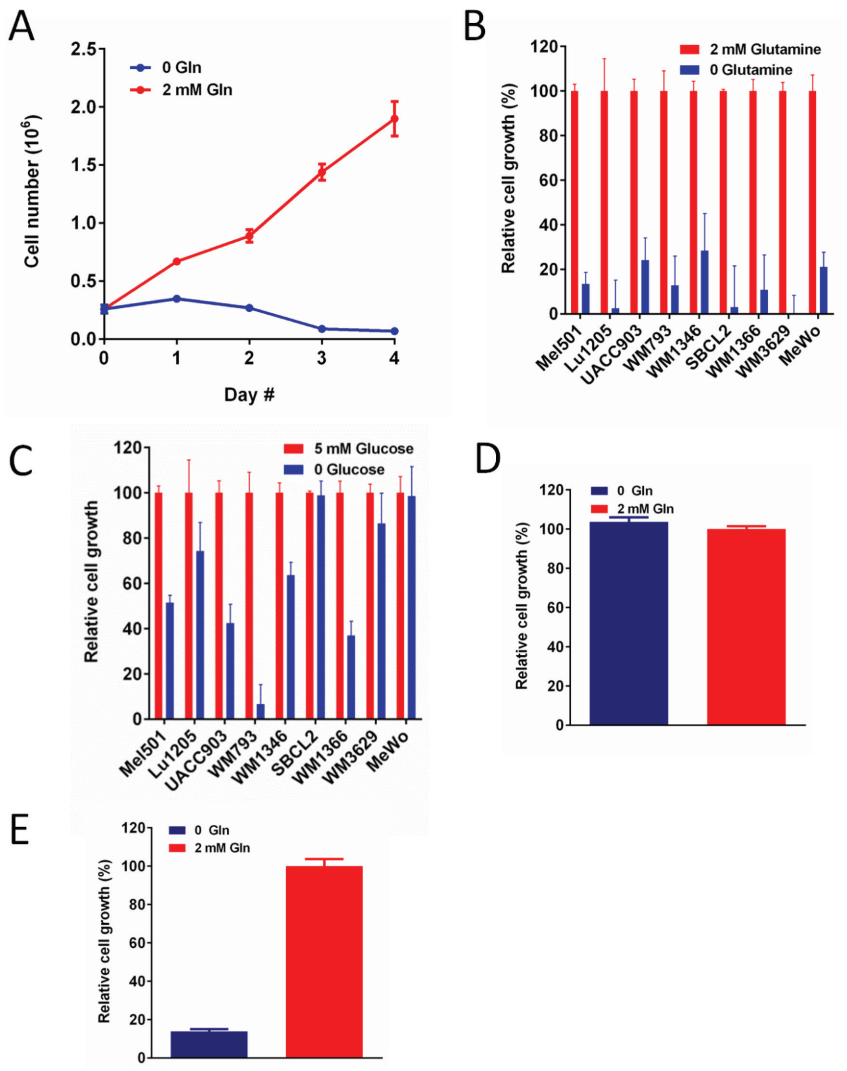


**Supplementary Figure S1: Melanoma cells need glutamine for growth and survival but melanocytes do not. (A)** Time course of growth of melanoma cell line Lu1205 in DMEM with or without 2 mM glutamine. **(B)** Growth of 9 melanoma cell lines including Lu1205 ± glutamine after 72 h. **(C)** Growth of melanoma cell lines with or without glucose, after 72 h. **(D)** Growth of primary human melanocytes in ATCC melanocyte medium ± glutamine. **(E)** Growth of Lu1205 melanoma cells in melanocyte medium ± glutamine. (Mean ± SEM of $N = 3$).

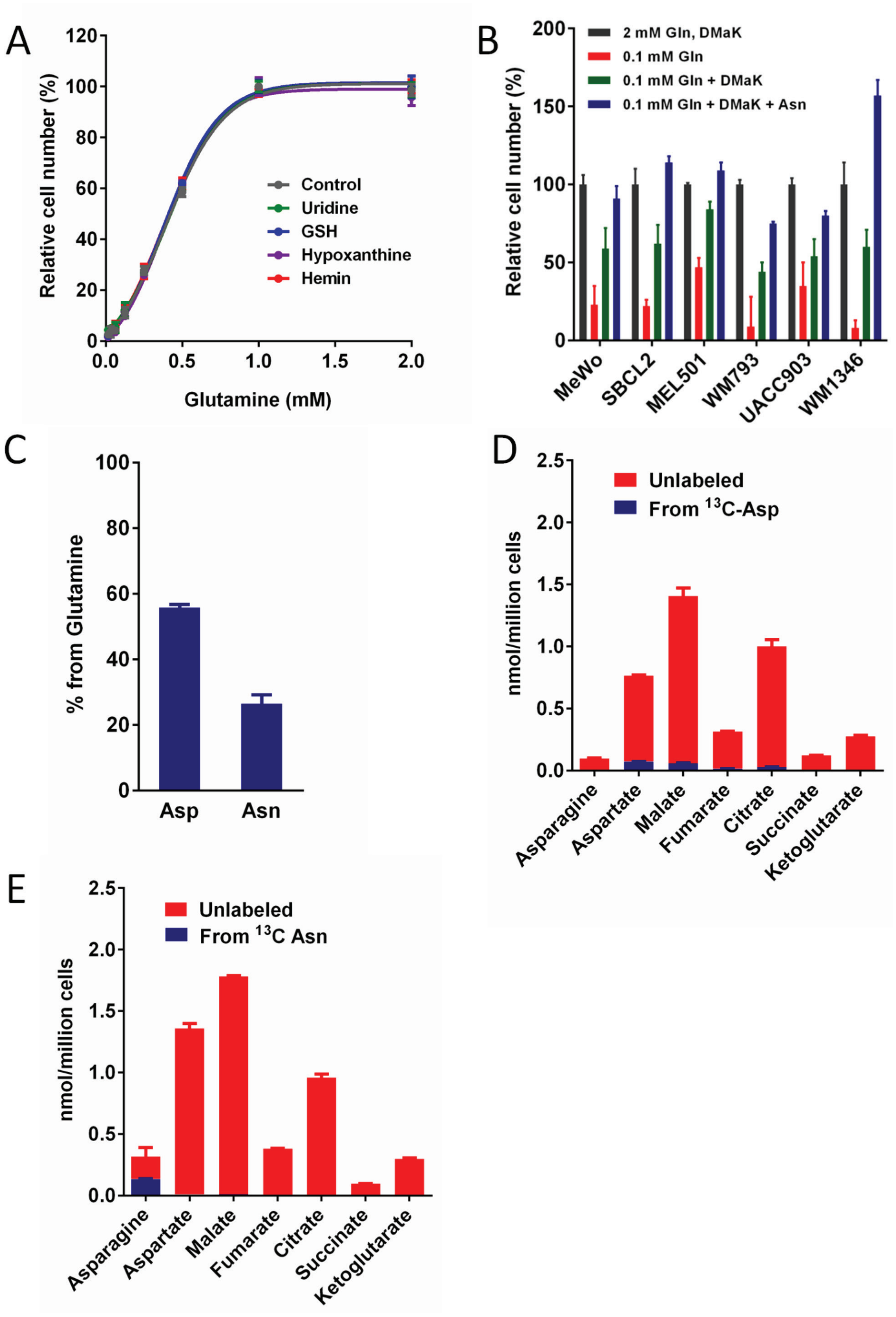


**Supplementary Figure S2: Alternative nutrients to replace glutamine, and aspartate and asparagine labeling.** **(A)** Uridine, glutathione, hypoxanthine and hemin do not reduce glutamine requirement for growth of Lu1205 cells. Lu1205 cells were grown in DMEM as in Figure 2A, and supplemented with uridine (20 µM), reduced glutathione (3 µM), hypoxanthine (30 µM), or hemin (15 µM). **(B)** Stimulation of growth of 6 melanoma cell lines in DMEM/0.1 mM glutamine by addition of 3 mM DMaK and/or 0.1 mM asparagine (growth relative to growth in DMEM with 2 mM glutamine and 3 mM DMaK). **(C)** Percent derivation of aspartate (Asp) or asparagine (Asn) from glutamine in Lu1205 cells labeled with $^{13}$C-glutamine. **(D)** Lu1205 cells were labeled with $^{13}$C-aspartate and amounts and $^{13}$C-labeling of cellular metabolites (asparagine, aspartate and TCA cycle metabolites) determined. Red + blue bars show total cellular metabolite amounts; blue bars alone indicate the fraction that was $^{13}$C-labeled. Medium aspartate was 98% $^{13}$C-labeled. **(E)** Lu1205 cells were labeled with $^{13}$C-asparagine, similarly to **(D)** Medium asparagine was 97% $^{13}$C-labeled. (Mean ± SEM of $N = 3$, except (C, E) ± range of $N = 2$).

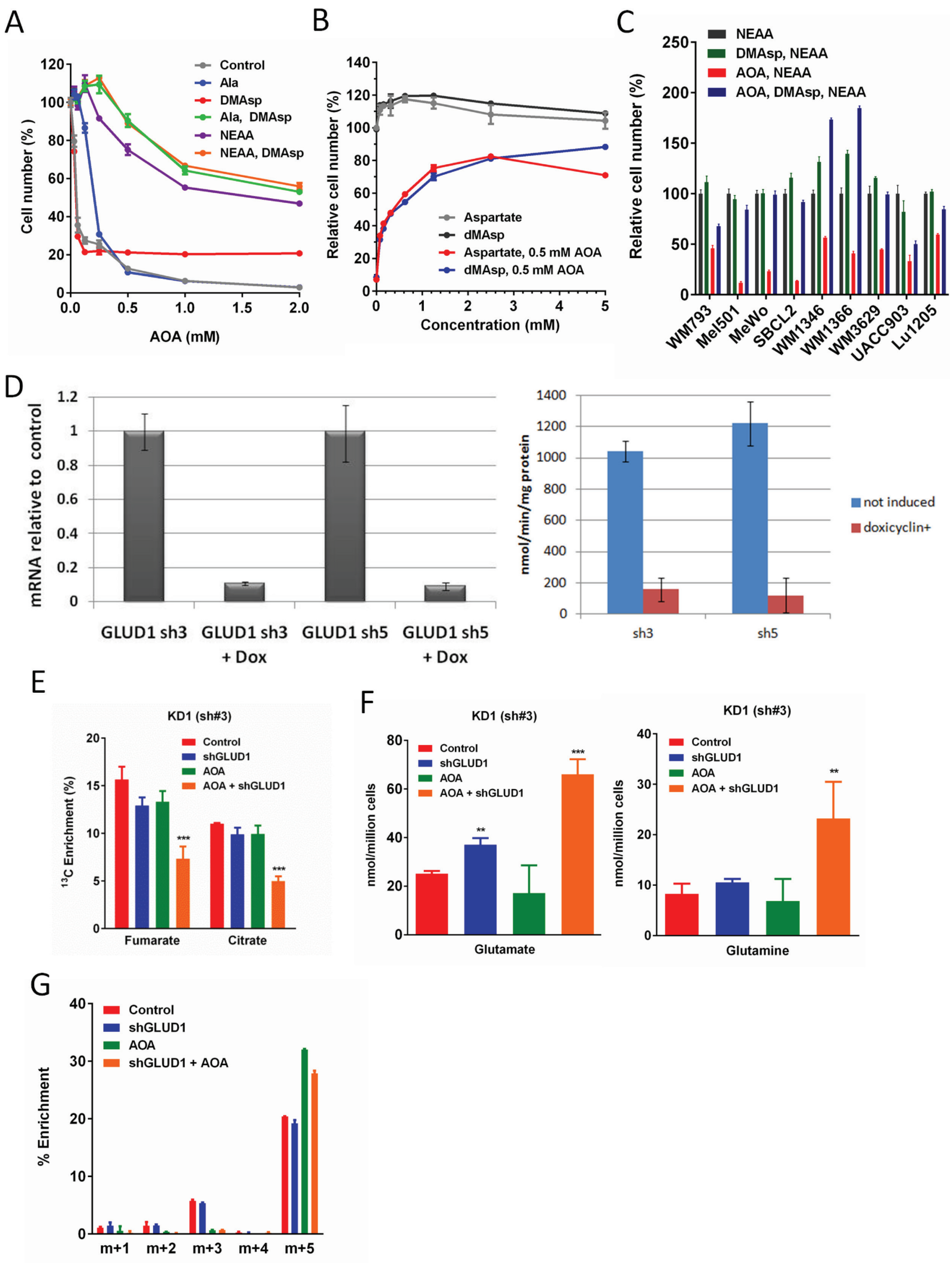


**Supplementary Figure S3: Effects of aminotransferase inhibition and glutamate dehydrogenase (GLUD1) knockdown.** **(A)** Inhibition of growth of Lu1205 cells by aminooxyacetate (AOA) in DMEM supplemented with 0.1 mM alanine, 5 mM dimethyl-aspartate (DMAsp), 0.1 mM NEAA, or combinations as shown. **(B)** Inhibition of growth of Lu1205 cells by AOA and reversal by aspartate or dimethyl-aspartate (dMAsp). Cells were treated for 72 h with 0.5 mM AOA and aspartate or dMAsp at the indicated concentrations. Medium also contained 0.1 mM alanine. **(C)** Comparative effects on growth of nine melanoma cell lines of 0.5 mM AOA treatment in medium with 2 mM glutamine (and 0.1 mM NEAA), with or without 5 mM DMAsp. **(D)** Relative mRNA levels of GLUD1 and glutamate dehydrogenase activity in non-induced or induced (with doxycycline, DOX) stable (shRNA) GLUD1-knockdown-modified Lu1205 cells, corresponding to this figure parts E-G (sh#3) or Figure 4B–4D (sh#5). **(E–G)** Effects of combined AOA treatment and GLUD1 knockdown on metabolism and growth using *GLUD1* construct sh#3. **(E)** Labeling of TCA cycle metabolites fumarate and citrate from $^{13}$C-glutamine. **(F)** Quantities of cellular free glutamate and glutamine. **(G)** Mass distribution of secreted glutamate (corrected for natural labeling and $^{13}$C-glutamine impurity). (Mean ± SEM of $N = 3$ for all, except mean ± range of $N = 2$ for **(G)**). $^{**}p < 0.05$, $^{***}p < 0.01$ by Student's unpaired *t*-test.

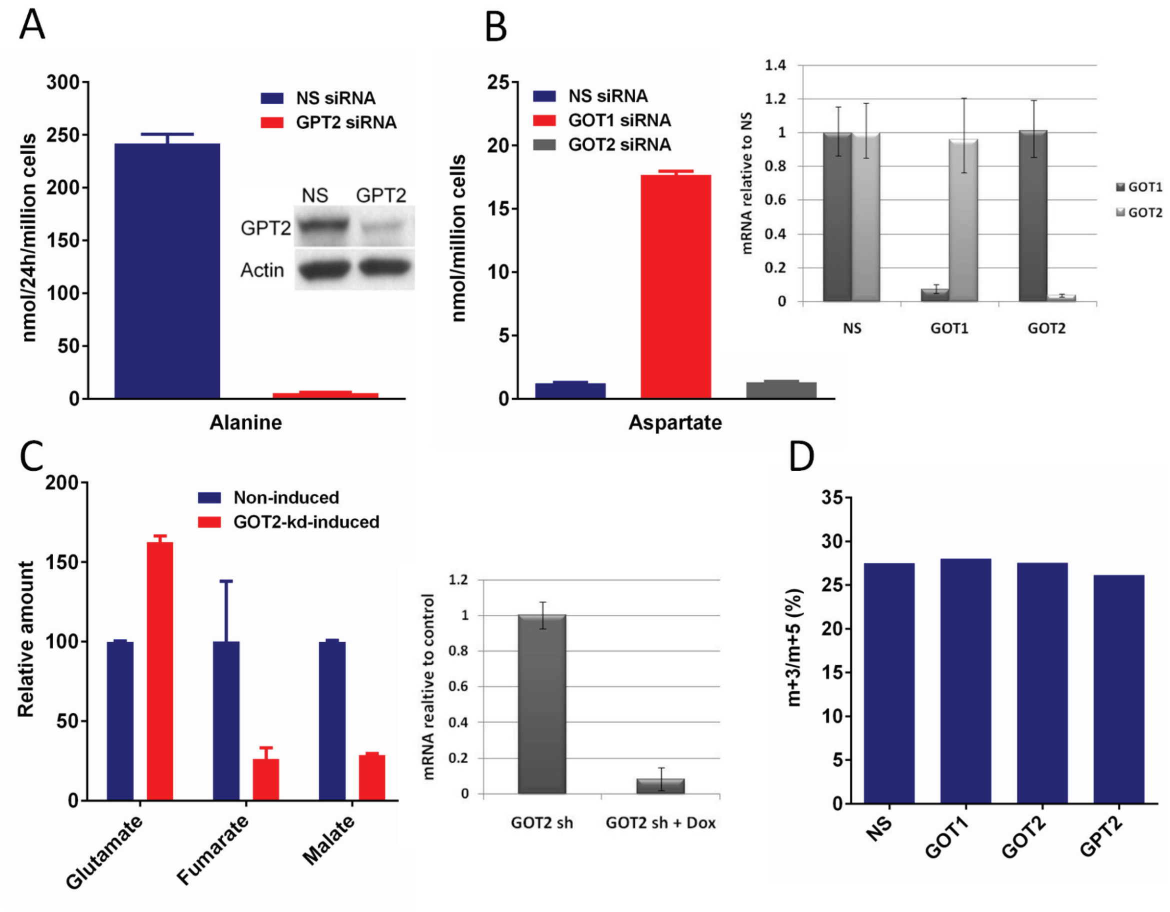


**Supplementary Figure S4: Metabolic effects of alanine aminotransferase or aspartate aminotransferase knockdown in Lu1205 cells.** **(A)** Effect of alanine aminotransferase (GPT2) siRNA knockdown on alanine secretion. Inset: Western blot for GPT2 in non-specific-siRNA (NS) and *GPT2*-specific-siRNA Lu1205 cells. Comparative control blot for β-actin is shown. **(B)** Cellular aspartate pools in control (NS) cells or cells following siRNA knockdown of *GOT1* or *GOT2* aspartate aminotransferases. Right panel: Relative mRNA levels of *GOT1* and *GOT2* in these cells. **(C)** Effect of *GOT2* knockdown on cellular free glutamate or the TCA cycle metabolites fumarate and malate. Right panel: Relative mRNA levels of *GOT2* in non-induced or induced (+DOX) shGOT2 -modified Lu1205 cells. **(D)** $^{13}$C-Labeling of extracellular glutamate from GOT1, GOT2 or GPT2 knockdown cultures, as in parts (A, B). Amount of m+3 glutamate (originating from TCA cycle) relative to m+5 glutamate (originating directly from deamidation of $^{13}$C-glutamine) is shown. (Data from representative experiments are shown. Error bars show range of technical replicates).
